# Short Communication: Quantification of the Effect of Mycotoxin Binders on the Bioavailability of Fat-Soluble Vitamins In Vitro

**DOI:** 10.3390/ani11082251

**Published:** 2021-07-30

**Authors:** Abdelhacib Kihal, María Ercilda Rodríguez-Prado, Carles Cristofol, Sergio Calsamiglia

**Affiliations:** 1 Animal Nutrition and Welfare Service, Departament de Ciència Animal i dels Aliments, Universitat Autònoma de Barcelona, 08193 Bellaterra, Spain; abdelhacib.khl@gmail.com (A.K.); maria.rodriguez.prado@uab.cat (M.E.R.-P.); 2 Servei d’Analisi de Fàrmacs, Departament de Farmacologia, de Terapèutica i de Toxicologia, Universitat Autònoma de Barcelona, 08193 Bellaterra, Spain; carles.cristofol@uab.cat

**Keywords:** mycotoxin binder, adsorption, fat-soluble vitamins

## Abstract

**Simple Summary:**

Mycotoxins are frequently found in animal feeds. Mycotoxicosis are often subclinical and difficult to diagnose, but result in important production losses. Mycotoxin binders are used to reduce their impact on health and performance. However, the unspecific mechanism of adsorption may also bind other essential nutrients, including vitamins. The aim of this study was to evaluate the effect of six mycotoxin binders on the bioavailability of fat-soluble vitamins by simulating the gastro intestinal digestion in vitro. The adsorption tests indicate that many mycotoxin binders adsorb a considerable proportion of vitamin E but not vitamin D, whereas the low recovery rate of vitamin A did not allow the quantification of its adsorption.

**Abstract:**

The aim of this study was to determine the capacity of six mycotoxin binders (MTBs) to adsorb vitamins A, D and E in an in vitro system that simulates gastric and intestinal digestion. Experiment 1 evaluated the recovery rate of vitamins A, D and E in the incubation conditions. In Experiment 2, the main factors were the MTB (bentonite, clinoptilolite, sepiolite, montmorillonite, active carbon and yeast cell walls), vitamins (A, D and E) and incubation type (vitamins incubated separately or together). The recovery was high for vitamin D (83%) and E (93%), but low for vitamin A (23%), for which no further analyses were conducted. When incubated separately, vitamin D was only adsorbed by yeast cell wall (20.2%). Vitamin E adsorption was highest with bentonite (54.5%) and montmorillonite (46.3%) and lowest with sepiolite (16.6%) and active carbon (18.5%). When incubated together, vitamin D was not adsorbed by any MTB. Vitamin E adsorption was highest in bentonite (61.8%) and montmorillonite (50.7%) and lowest in sepiolite (15.4%). Results indicate that the bioavailability of vitamin E, but not that of vitamin D, may be reduced in the presence of MTBs.

## 1. Introduction

Mycotoxin occurrence in feeds is increasing and influenced by the effect of climate change. Recent reports indicate that up to 65% of food samples analyzed worldwide are contaminated with at least one mycotoxin [1,2]. Mycotoxicoses cause considerable production and economic losses [3]. Mycotoxin binders (MTBs) are designed to sequester different mycotoxins, although their adsorption effectiveness varies depending on the binder and the mycotoxin [4,5,6]. Mycotoxin binding is based on weak anionic–cationic interactions between the MTB and the toxin and differences in size, structure and charge of the binding sites determine the capacity of binders to adsorb different molecules. This unspecific mechanism of action allows other molecules to be bound similarly. The European Food Safety Authority (EFSA) [7] requires in vitro tests to be conducted to evaluate the potential sequestration of essential nutrients by MTBs, including vitamins A (VA) and E (VE). Previous studies reported that some MTBs adsorb relevant proportions of vitamins B1 and B6 [8,9]. In contrast, the few experiments conducted in vitro to evaluate the capacity of MTBs to adsorb vitamins D (VD) and VE reported that the binding capacity is low [10]. Surprisingly, and in spite of the requirements of the EFSA [7], we found no reports on the interactions between MTBs and VA. Overall, there is very limited information on the potential interaction between MTBs and fat-soluble vitamins (FSVs).

We hypothesized that the adsorption of FSVs by MTBs is high. The aim of this study was to determine the capacity of six mycotoxin binders to adsorb FSVs in vitro.

## 2. Materials and Methods

### 2.1. Experimental Design and Incubations

The in vitro incubation was an adaptation of the technique described by Lemke et al. [11] and Gallo and Masoero [12]. The gastric digestion model was prepared with 1.25 g/L of pepsin (CAS: 9001-75-6, 77160, Sigma, St. Louis, MO, USA), 0.5 mL of malic acid (CAS: 6915-15-7, M0875, Sigma), 42 µL/L of lactic acid (CAS: 50-21-5, 252476, Sigma), 0.5 g/L of citric acid (CAS: 5949-29-1, 141018, Panreac AppliChem GmbH, Darmstadt, Germany) and 50 µL/L of acetic acid (CAS: 64-19-7, 131008, Panreac AppliChem GmbH), and adjusted to pH 3.0 with hydrochloric acid. Tubes were incubated in a water bath at 37 °C for 2 h and shaken with a vortex at the start of the incubation and at 1-h intervals until the end of the incubation. After 2 h, the pH was neutralized at 6.5 with a sodium bicarbonate solution (8.8 g/100 mL) and a second buffer solution containing 3.5 g/100 mL of bile salts (CAS: 110-86-1, 48305, Sigma) and 1 g/100 mL of pancreatin (CAS: 8049-47-6, P7545, Sigma) to simulate the intestinal digestion. The incubation continued under the same conditions for two additional hours. Vitamins used were: vitamin A (retinol palmitate, CAS: 79-81-2, PHR1235, Sigma); vitamin D, (cholecalciferol, CAS: 67-97-0, C9756, Sigma) and vitamin E (DL-α-Tocopherol, CAS: 7695-91-2, T3376, Sigma). The mycotoxin binders used were: bentonite, clinoptilolite, sepiolite, montmorillonite (MMT), active carbon (AC) and yeast cell wall (YCW).

A preliminary test was conducted to evaluate the stability of FSVs in the in vitro gastrointestinal model before assessing their adsorption by MTBs. Incubations were conducted in triplicate and in two consecutive periods. Vitamins (5 µg/mL) were incubated in 15 mL glass tubes containing 2 mL of the gastric digestion solution and after 2 h, the pH was neutralized at 6.5 with bicarbonate solution (8.8 g/100 mL) and 80 µL of the intestinal digestion solution were added. Each vitamin was prepared in an ethanol solution just before the test with minimal exposure to air and light. Samples were taken for analysis at 0, 1, 2, 3 and 4 h of incubation. For each incubation time, samples were incubated separately in triplicate to avoid sub-sampling for each time and to preserve the whole amount of the incubated vitamins for analysis.

The adsorption study was designed as a 6 × 3 × 2 factorial being MTBs, FSVs and the type of incubation (incubations of each MTB with each vitamin separately or each MTB with the three FSVs together) the main factors. Mycotoxin binders were dosed at 2 mg/mL. Vitamin doses were calculated to maintain the MTB:vitamin ratio within physiological conditions [9,13], and included 5 µg/mL of VA, 0.15 µg/mL VD and 149 µg/mL of VE. All incubations included a blank consisting of the buffer with neither MTBs nor vitamins, and a positive control with buffer and each vitamin but not MTBs. Incubations were conducted in triplicate within a period and in two independent periods. Samples were incubated in 100 mL tubes containing 50 mL of the gastric digestion solution and 2 mL of the intestinal digestion solution.

At the end of the incubation period, 0.5 mL sub-samples of each individual tube were placed in 1.5 mL Eppendorf tubes with 0.5 mL of ethanol. Samples were shaken in a multi-vortex for 3 min followed by centrifugation at 23,000× *g*, at 4 °C for 10 min. A volume of 500 µL of the supernatant was placed in a 2 mL HPLC glass vial for analysis. Exposure to air or light were carefully limited throughout the process.

The quantification of the FSVs was performed with an HPLC-UV technique. An Agilent 1100 system (Darmstadt, Germany) equipped with an autosampler (G1330A, Darmstadt, Germany), a quaternary pump (G1311A, Darmstadt, Germany), and a UV detector (G1315A, Darmstadt, Germany) were used for the FSV quantification. The obtained data analysis was performed using an IBM computer with ChemStation software (Agilent Technologies, Santa Clara, CA, USA). To separate the FSVs, an Infinity Lab Poroshell 120 SB-18 (2.1 × 100, 2.7 µm) column (Agilent Technologies, Santa Clara, CA, USA) was used. The flow rate was 0.5 mL/min and the mobile phase was composed by acetonitrile (A) and a 0.1% phosphoric acid water solution (B) in a gradient of solvents as follows: The initial conditions were 50% A and 50% B; for 2 min, the conditions changed to 100% of A and this proportion was maintained for 7 min. Finally, it returned to initial conditions in 0.1 min. The total runtime of the analysis was 14 min. The FSVs were detected at 285 nm. The injection volume was 2 µL and the column was maintained at 50 °C. With these conditions, the retention times of VD, VE and VA were, 6.3, 7.3 and 10.7 min, respectively. The range of the calibration curves were 0.05 to 20 µg/mL and the limit of quantification was considered as the lower level of the calibration curve.

### 2.2. Calculations and Statistical Analysis

The adsorption index (AI) was calculated as the ratio between the difference of the concentration of each substrate in the positive control without MTB and the corresponding treatment concentration of the substrate incubated with the MTB, vs. the concentration in the positive control as: AI = (Ci − Ct) × 100/Ci, where Ci = initial concentration of the positive control (mg/mL), and Ct = treatment concentration of the unbound substrate with MTB (mg/mL) from the corresponding positive control of the substrate studied. The AI was expressed as a percent of the substrate bound to the mycotoxin binder.

Vitamin adsorption results were analyzed using the PROC MIXED procedure of SAS (v.9.4; SAS Institute, Inc., Cary, NC, USA). The model included the MTBs, the FSVs, the type of incubation and their interactions as fixed effects, and the experimental period as a random effect. Results are presented as least squares means. When differences among means were significant (*p* < 0.05), means were separated with the Tukey test using the SLICEBY option of the PROC PLM of SAS.

## 3. Results

**Figure 1:**
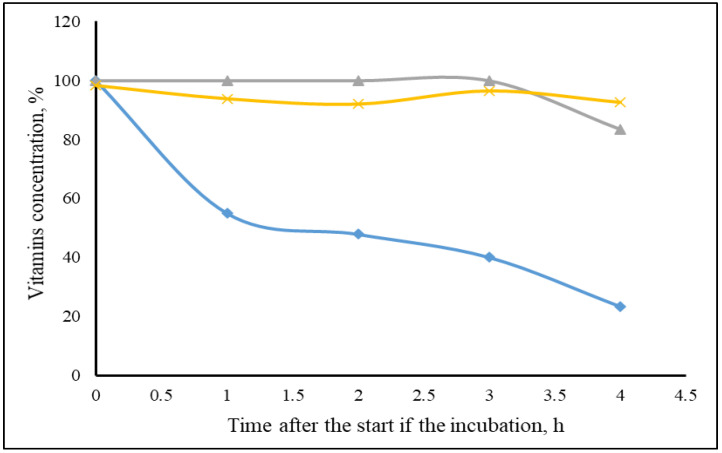
Recovery rate of vitamin A (♦), vitamin D (▲) and vitamin E (×) in in vitro gastro intestinal digestion method.

In the preliminary test, the recovery of FSVs as a percentage of the original concentration is depicted in Figure 1. Concentrations of VD and VE were constant in the first 3 h with 100 and 95% recovery rate, respectively, and at the end of the incubation, this slightly decreased to 83 and 93%, respectively. In contrast, VA recovery progressively decreased over time to 50% at 2 h and decreased to only 23% after 4 h. Therefore, the adsorption capacity of MTBs on VA could not be evaluated.

For the adsorption study, when vitamins were incubated separately, overall, VD had lower adsorption than VE (average of 6.6 vs. 33.3%, respectively; *p* < 0.01). The AI of each vitamin with each MTB when incubated separately is shown in Table 1. The adsorption of VD was only small but significant with YCW (20.2%; *p* < 0.01), tended to be different from 0 in sepiolite and AC (8.6 and 8.5%, respectively; *p* < 0.07) and was not different from zero in all other MTBs (*p* > 0.18). In contrast, VE adsorption was highest with bentonite and MMT (average of 50.4%), intermediate with clinoptilolite and YCW (average of 32.0%) and lowest with sepiolite and AC (average of 17.6%). The AI of each vitamin in each adsorbent when incubated together is shown in Table 1.

When vitamins were incubated together, VD was not adsorbed by any of the MTBs (*p* < 0.05). In contrast, VE was adsorbed in similar amounts when incubated separately or together (average of 36.3 vs. 33.4%, respectively; *p* < 0.18), ranging from 61.8 to 16.6% for bentonite and sepiolite, respectively. 

## 4. Discussion

Fat soluble vitamins are very sensitive to light, oxygen, temperature and pH exposure. Because the AI was calculated based on the remaining vitamin in the incubation medium compared with the positive control, it was important to confirm the stability of vitamins during the four hours of incubation. Vitamins D and E were relatively stable. However, the degradation of VA was very high and progressive with time to the point that only 25% of the initial VA was recovered after the 4 h incubation, in spite of all precaution measures taken to minimize exposure to air and light throughout the experiment. The temperature (37 °C) and pH (3.0 and 6.5) were intrinsic to the method and could not be modified. Surprisingly, and in spite of the EFSA [7] requiring testing of the interaction between MTB and VA for approval, we found no reports in literature describing this interaction. Although it appears that adsorption of vitamins by MTBs occurs within the first 5 min of incubation (data not shown), the high VA degradation with time using this in vitro method makes the evaluation of the impact of MTBs on VA adsorption unfeasible. Therefore, no further tests were conducted with VA.

Negative values of VD were not different from zero and reflected lack of adsorption. When VD was incubated alone, adsorption was relatively small and only significant in YCW, whereas adsorption by other MTBs was either quantitatively small for sepiolite and AC, or negligeable for the rest of MTB. These results are consistent with previous research that reported that bentonite did not adsorb VD in an in vitro simulated gastrointestinal digestion test [10]. The interaction of substrates with MTB is due to weak ionic bonds between charged groups of the vitamin and the cations presents in the interlayer space of clays. The large molecular weight (384.6 g/mol) and the structure of VD that includes 27 carbons with two aromatic cycles may compromise the capacity of the vitamin to physically get into the interlayer space of clays or the pores of AC, resulting in a low adsorption. In contrast, the adsorption mechanism of YCW is different from that of clays. The β-D-glucans of YCW play the main role in the adsorption mechanism and are located in its external surface. There, the interaction of the carboxyl group and the aromatic rings of VD with β-D-glucans structure are more dependent on the three-dimensional match of the molecules and less on its size or molecular weight itself [14]. However, a good match is more difficult in complex molecular structures like VD, with several aromatic rings, which may explain the relatively low adsorption (20.2%). When VD was incubated with the other vitamins, there was no adsorption by any of the MTB. The presence of the three vitamins together may create competition among them for the adsorption site of MTB. Therefore, it is likely that the high affinity of VE for YCW when incubated with other vitamins (23.7%) displaced VD from the binding site, reducing its adsorption from 20.2% when incubated alone to −1.0% when incubated together with VE.

When vitamin E was incubated alone, the adsorption capacity ranged from 16.6 to 54.5%, with the highest adsorption observed in bentonite and MMT. This result is similar to previous studies with water-soluble vitamins, where bentonite and MMT had the highest affinity to adsorb vitamins B1, B2 and B6 [9]. Although VE has a molecular weight higher than that of VD (430 vs. 384 g/mols, respectively), the straight flat molecular structure with one carboxyl group and a single aromatic cycle may favor the interaction with the interlayer space of MTBs. The molecular structure of MTBs plays an important role in determining the adsorption capacity of each binder. For instance, the interlayer space of clay binders depends on the clay type and source. Mortland and Lawless [15] reported that Na^+^ smectite has a higher capacity to adsorb vitamin B2 than Ca^2+^ smectite and attributed this difference to the larger interlayer space for Na^+^ (~20 Å) compared with Ca^2+^ (~15.0 Å) smectite. It is suggested that bentonite and MMT have a larger interlayer space than the other MTBs tested and allows the VE to get into it. In contrast, adsorption of VE by sepiolite and AC was the lowest when incubated alone. Vitamins B1 and B6 were highly adsorbed by sepiolite, but their molecular weight (about half that of VE) and structure was more adequate for adsorption. The interaction of vitamins with AC is different because the pore size is measured in nanometers, which is much larger than the size of the interlayers of clays, measured in amstrongs. Therefore, the possibility of stabilizing bonds is more difficult in the AC pores, which may explain the lower affinity.

The absorption of VE by MTBs was not affected by the presence of other vitamins in the mix (average of 36.3 vs. 33.4% when incubated alone or together with other vitamins, respectively; *p* < 0.18). Vitamin E adsorption ranged from 16.6% in sepiolite to 61.8% in bentonite. Results indicate that the affinity of VE for MTBs is higher than that of VD, and the higher affinity of VE for YCW (30.0 and 23.7% when incubated alone or mixed, respectively) displaced VD (20.2 and −1.0% when incubated alone or together, respectively). The interpretation of our results is that there is competition among vitamins for binding sites, and that this competition may also occur with mycotoxins, which, in turn, may reduce the adsorption of mycotoxins or the bioavailability of some essential nutrients.

## 5. Conclusions

Results indicate that the bioavailability of vitamin E, and to a much lesser extent vitamin D, may be reduced significantly by mycotoxin binders. The results also suggest that when mixed together, vitamin E may displace vitamin D from the binding sites of mycotoxin binders, suggesting possible competition among molecules for the binding sites.

## Figures and Tables

**Table 1:** Adsorption index of the six mycotoxin binders with vitamins D and E when incubated separately or together.

Vitamins	Mycotoxin Binders					
	Bentonite	Clinoptilolite	Sepiolite	MMT ^1^	AC ^2^	YCW ^3^
Incubated separately						
D	−4.4 ^z,c^	2.9 ^z,b^	8.6 ^b^	5.2 ^z,b^	8.5 ^b^	20.2 ^a^
E	54.5 ^y,a^	34.1 ^y,b^	16.6 ^c^	46.3 ^y,a^	18.5 ^c^	30.0 ^b^
SEM ^4^ 1	5.88					
Incubated together						
D	−2.5 ^z,b^	−2.5 ^z,b^	−0.3 ^z,b^	7.4 ^z,a^	−2.9 ^z,b^	−1.0 ^z,b^
E	61.8 ^y,a^	38.3 ^y,b^	16.6 ^y,d^	50.7 ^y,a^	28.5 ^y,bc^	23.7 ^y,cd^
SEM 2	3.54					

^a–c^ Different letters in the same row indicate a significant effect between binders (*p* < 0.05). ^y,z^ Different letters in the same column and type of incubation indicate significant effect between vitamins (*p* < 0.05). ^1^ MMT: montmorillonite. ^2^ AC: active carbon. ^3^ YCW: yeast cell wall. ^4^ SEM: standard error of the mean.

## Data Availability

Raw data available at doi:10.17632/3trpspy2xm.1.
